# Three new indole alkaloids from *Rauvolfia yunnanensis*

**DOI:** 10.1007/s13659-011-0023-7

**Published:** 2011-12-01

**Authors:** Yuan Gao, Fei Wang, Dong-Sheng Zhou, Yan Li, Ji-Kai Liu

**Affiliations:** 1 State Key Laboratory of Phytochemistry and Plant Resources in West China, Kunming Institute of Botany, Chinese Academy of Sciences, Kunming, 650201 China; 2 BioBioPha Co., Ltd., Kunming, 650201 China; 3 Graduate University of Chinese Academy of Sciences, Beijing, 100049 China

**Keywords:** *Rauvolfia yunnanensis*, indole alkaloid, rauvoyunine

## Abstract

One rare tetracyclic macroline-type indole alkaloid, named rauvoyunine A (**1**), and two new picraline-type alkaloids rauvoyunines B and C (**2** and **3**) were isolated from the aerial parts of *Rauvolfia yunnanensis*. Their structures were elucidated on the basis of extensive spectroscopic analysis. Compounds **2** and **3** were evaluated for their *in vitro* cytotoxicity against five human tumor cell lines.

**Electronic Supplementary Material:**

Supplementary material is available for this article at 10.1007/s13659-011-0023-7 and is accessible for authorized users.

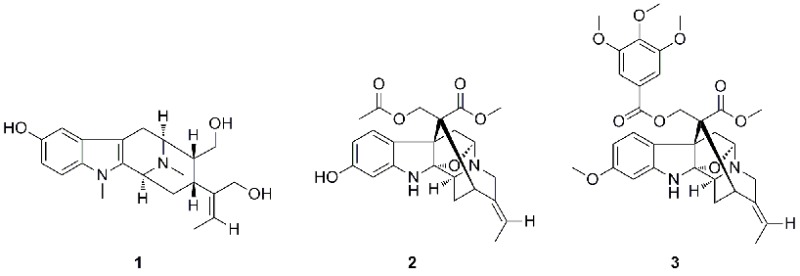

## Introduction

The plants of genus *Rauvolfia*, widely distributed in America, Africa, Asia, and Oceania,[1] are well-known rich sources of unique heterocyclic alkaloids with monoterpene indole skeletons. These alkaloids have attracted great interests from biological and therapeutic aspects, due to their anticancer,[2] antimalarial,[3] antihypertensive,[4] and sedative[5] properties.

*Rauvolfia yunnanensis* Tsiang, belonging to the Apocynaceae, is indigenous to southwestern China, and its roots are utilized locally for the remedy of scab[1]. Recently, several structurally interesting indole alkaloids were isolated from this plant.[6–8] As one part of our research program exploring bioactive monoterpene indole alkaloids from Chinese species of *Rauvolfia*, phytochemical analysis from the methanol extract of aerial parts of *R. yunnanensis* led to the isolation of three new alkaloids, rauvoyunines A–C (**1**–**3**). This paper reports the isolation and structure elucidation of new compounds and cytotoxic evaluation of selected compounds.

## Results and Discussion

Compound **1**, obtained as amorphous powder, has a molecular formula of $C_{21}H_{28}N_2O_3$ based on HRESIMS (pos.), showing a quasi-molecular ion peak at *m/z* 357.2185 (calc. for $C_{21}H_{29}N_2O_3$, 357.2178). The UV spectrum showed absorptions at $\lambda_{max}$ 228, 282, and 303 nm typical of a substituted indole chromophore.[9] The IR spectrum showed absorptions at 3432 and 1628 $cm^{-1}$, attributed to hydroxy and olefin groups, respectively. The $^1H$ NMR spectrum (Table 1) showed signals for an aromatic AMX spin system at $\delta_H$ 6.73 (dd, $J$ = 8.7 and 2.3 Hz), 6.85 (d, $J$ = 2.3 Hz), and 7.17 (d, $J$ = 8.7 Hz), typical of an indole moiety substituted by a hydroxy group at 10 or 11 position, signals characteristic of an ethylidene group at $\delta_H$ 1.44 (d, $J$ = 6.8 Hz) and 5.58 (q, $J$ = 6.8 Hz), singlets for N-methyl groups at $\delta_H$ 2.55 and 3.59, and resonances of two hydroxymethyl groups, one at $\delta_H$ 3.75 (dd, $J$ = 10.5 and 3.7 Hz) and 3.91 (dd, $J$ = 10.5 and 5.5 Hz), and another at $\delta_H$ 4.02 (2H, s). The $^{13}C$ NMR (DEPT) spectrum (Table 1) exhibited six $sp^2$ quaternary carbons ($\delta_C$ 151.8, 140.3, 134.0, 132.3, 127.8, and 106.2), four $sp^2$ methines ($\delta_C$ 124.8, 112.2, 110.6, and 103.4), four $sp^3$ methines ($\delta_C$ 58.7, 55.5, 47.7, and 30.2), four $sp^3$ methylenes ($\delta_C$ 66.0, 64.1, 30.5, and 23.7), and three methyl groups ($\delta_C$ 41.5, 29.3, and 13.1). The above-mentioned data, together with the presence of nine degrees of unsaturation suggested that **1** was an indole alkaloid with tetracyclic macroline skeleton.[10,11] This conclusion is supported by the HMBC (Figure 1) three-bond correlations of H-5 to C-3, C-15, and C-17, H-16 to C-6 and C-14, and H-15 to C-3, C-17, and C-19. In addition, the *m*-coupling doublet at $\delta_H$ 6.85 ($J$ = 2.3 Hz) showed an HMBC correlation with C-7 and a ROESY correlation with H-6$\beta$, indicating the hydroxyl at C-10. HMBC cross-peaks of H-18 to C-19 and C-20, and H-19 to C-15 and C-20 located the ethylidene side chain at C-20, and cross-peaks of H-17 to C-5, C-15, and C-16, and H-21 to C-15, C-19, and C-20 revealed that the two hydroxymethyl group was adjacent to C-16 and C-20, respectively. The position of each methyl group was confirmed by HMBC correlations from N(1)-Me to C-2 and C-13, and N(4)-Me to C-3 and C-5.

The relative stereochemistry of **1** was elucidated by ROESY experiments (Figure 1). The ROESY spectrum exhibited correlations of H-16↔H-6$\beta$ and H-3↔N(4)-Me↔H-5, fixing the two bridgehead hydrogen atoms as $\alpha$ orientation, revealing

*To whom correspondence should be addressed. E-mail: jkliu@mail.kib.ac.cn

**1**

**2**

**3**

the stereochemistry of H-16 as *β*-oriented. The ROESY correlation between H-14*α* and the hydroxymethyl proton at C-20 indicated that C-20 was *α*-oriented and H-15 was *β*-oriented. The ROESY correlations of Me-18↔H-15 and H-19↔H-21 confirmed that the ethylidene side chain had an *E*-configuration. Consequently, the structure of compound **1** was determined as shown, named rauvoyunine A.

**Figure 1.** Key HMBC and ROESY correlations of **1**.

Compound **2** was obtained as amorphous powder. Its molecular formula was determined to be $C_{23}H_{26}N_2O_6$ on the basis of HRESIMS (pos.), showing a quasi-molecular ion peak at *m*/*z* 427.1868 (calc. for $C_{23}H_{27}N_2O_6$, 427.1869). The UV spectrum showed maximum absorption at 292 nm, and the IR spectrum implied the presence of carbonyl (1738 $cm^{-1}$) functionality. The $^1H$ NMR spectra (Table 1) showed the presence of three aromatic protons, an ethylidene side chain, a methyl ether, and an acetyl. The $^{13}C$ NMR spectra (Table 1) revealed 23 carbon signals due to six $sp^2$ quaternary carbons, four $sp^2$ methines, three $sp^3$ quaternary carbons, three $sp^3$ methines, four $sp^3$ methylenes, and three methyl groups. These spectroscopic features suggested that **2** had a picraline-type skeleton.[13,14] The three observed aromatic resonances at $\delta_H$ 7.11 (d, $J$ = 8.8 Hz), 6.20 (dd, $J$ = 8.8, 2.2 Hz), and 6.19 (d, $J$ = 2.2 Hz) combined with the fact that a hydroxylated carbon signal was detected at $\delta_C$ 158.6 (s) suggested that an OH group located at C-10 or C-11. Finding HMBC correlation from the *o*-coupling doublet at $\delta_H$ 7.11 ($J$ = 8.8 Hz) to C-7 at $\delta_C$ 53.2 (s) and ROESY correlation between the doublet and H-6*α* further confirmed placement of hydroxyl at C-11. Two geminally coupled proton signals at $\delta_H$ 3.85 and 4.52 (each d, $J$ = 11.1 Hz) showed correlations with an *O*-acetyl carbonyl carbon at $\delta_C$ 171.9, indicating that an acetoxy group was located at C-17. An methoxy singlet at $\delta_H$ 3.66 showed correlation to C-22 at $\delta_C$ 173.7, allowing assignment of methyl ether at C-22 position. The ROESY cross-peak between H-17a and H-14*β* indicated that C-17 was *β*-oriented, which was further supported by the presence of an NMR signal of the acetyl methyl group at $\delta_H$ 1.62 (the noticeable upfield shift being due to the shielding by the aromatic ring current).[15] ROESY cross-peaks of H-14*α*↔H-21*α*↔H-3 and H-21*β*↔H-5 revealed that the configuration of H-3 and H-5 were *α* and *β*, respectively. The ethylidene moity was assigned to be *E*-type since the correlations of H-18↔H-15 and H-19↔H-21 were observed. Therefore, the structure of **2** was unambiguously elucidated as 11-hydroxy-picraline, named rauvoyunine B.

Compound **3**, obtained as amorphous powder, possessed a molecular formula of $C_{32}H_{36}N_2O_9$, as evidenced by HRESIMS (pos.) at *m*/*z* 593.2496 (calc. for $C_{32}H_{37}N_2O_9$, 593.2499), in combination with $^1H$ and $^{13}C$ NMR (DEPT) spectra (Table 1), and appropriate for 16 degrees of unsaturation. The UV spectrum showed maximum absorption at 269 nm. Its IR spectrum revealed absorption bands of carbonyl (1722 $cm^{-1}$). The $^1H$ NMR data showed signals of three aromatic protons at $\delta_H$ 6.04 (br. d, $J$ = 8.3 Hz), 6.26 (br. s), and 7.29 (d, $J$ = 8.3 Hz), an ethylidene group at $\delta_H$ 1.62 (d, $J$ = 6.7 Hz) and 5.48 (q, $J$ = 6.7 Hz), two methoxy groups at $\delta_H$ 3.45 (s) and 3.64 (s), and a 3′,4′,5′-trimethoxybenzoyloxy unit, based on the presence of two aromatic protons at $\delta_H$ 6.94 (2H, s) and three overlapped methoxy groups at $\delta_H$ 3.87 (9H, s). The $^{13}C$ NMR (DEPT) data exhibited ten $sp^2$ quaternary carbons, six $sp^2$ methines, three $sp^3$ quaternary carbons, three $sp^3$ methines, four $sp^3$ methylenes, and six methyl groups. These spectroscopic features were closely related to those of **2**, but it was evident that **3** had a trimethoxybenzoyloxy unit instead of the acetoxy moity at C-17 and a methoxyl group instead of the hydroxy group at C-11. This was also supported by HMBC correlations from H-17 to C-7′ and correlation from methoxy protons at $\delta_H$ 3.45 (s) to C-11 at $\delta_C$ 160.1. The ROESY cross-peaks of Me-18↔H-15 and H-19↔H-21 confirmed that the ethylidene possessed an *E*-configuration. The cross-peak of 17a↔H-14*β* indicated that the stereochemistry of C-16 was identical to that of **2**. Since the $J_{3\ 14}$, $J_{14\ 15}$, and $J_{5\ 6}$ values were essentially unchanged compared to **2**, it is safe to deduce that the ring junction stereochemistry remain intact. Thus, the structure of **3** was established to be 11-methoxyburnamine-17-*O*-3′,4′,5′-trimethoxybenzoate and named as rauvoyunine C.

Compounds **2** and **3** were evaluated for their cytotoxicities against five human cancer cell lines (HL-60, SMMC-7721, A-549, MCF-7, and SW-480) using the MTT method as

**Table 1.** $^1H$ **NMR and** $^{13}C$ **NMR spectroscopic data for rauvoyunines A−C (1−3) (*δ* in ppm, *J* in Hz).**

| No. | **1**[a] | | **2**[a] | | **3**[b] | |
|---|---|---|---|---|---|---|
| | $\delta_H$ | $\delta_C$ | $\delta_H$ | $\delta_C$ | $\delta_H$ | $\delta_C$ |
| 2 | | 132.3 (s) | | 109.0 (s) | | 107.5 (s) |
| 3 | 4.39 (dd, 4.2, 2.4) | 55.5 (d) | 3.48 (br. d, 3.1) | 52.7 (d) | 3.98 (br. s) | 51.9 (d) |
| 5 | 3.72 (d, 7.2) | 58.7 (d) | 4.72 (d, 2.8) | 88.1 (d) | 5.15 (br. s) | 87.4 (d) |
| 6α | 3.35 (dd, 16.6, 7.2) | 23.7 (t) | 2.29 (dd, 14.2, 2.8) | 45.0 (t) | 2.52 (br. d, 14.7) | 42.3 (t) |
| 6β | 2.63 (d, 16.6) | | 3.17 (d, 14.2) | | 3.17 (d, 14.7) | |
| 7 | | 106.2 (s) | | 53.2 (s) | | 51.9 (s) |
| 8 | | 127.8 (s) | | 126.1 (s) | | 124.6 (s) |
| 9 | 6.85 (d, 2.3) | 103.4 (d) | 7.11 (d, 8.8) | 128.6 (d) | 7.29 (d, 8.3) | 127.4 (d) |
| 10 | | 151.8 (s) | 6.20 (dd, 8.8, 2.2) | 108.0 (d) | 6.04 (br. d, 8.3) | 106.3 (d) |
| 11 | 6.73 (dd, 8.7, 2.3) | 112.2 (d) | | 158.6 (s) | | 160.1 (s) |
| 12 | 7.17 (d, 8.7) | 110.6 (d) | 6.19 (d, 2.2) | 99.5 (d) | 6.26 (br. s) | 98.2 (d) |
| 13 | | 134.0 (s) | | 151.7 (s) | | 149.1 (s) |
| 14α | 2.58 (ddd, 13.7, 13.3, 4.2) | 30.5 (t) | 2.00 (ddd, 15.1, 4.0, 3.1) | 22.5 (t) | 2.11 (2H, s) | 21.0 (t) |
| 14β | 1.73 (ddd, 13.7, 4.0, 2.4) | | 1.95 (br. d, 15.1) | | | |
| 15 | 2.98 (ddd, 13.3, 5.6, 4.0) | 30.2 (d) | 3.36 (br. s) | 36.3 (d) | 3.46 (br. s) | 34.5 (d) |
| 16 | 1.86 (ddd, 5.6, 5.5, 3.7) | 47.7 (d) | | 57.4 (s) | | 56.6 (s) |
| 17a | 3.91 (dd, 10.5, 5.5) | 64.1 (t) | 4.52 (d, 11.1) | 68.2 (t) | 4.61 (d, 11.2) | 66.7 (t) |
| 17b | 3.75 (dd, 10.5, 3.7) | | 3.85 (d, 11.1) | | 4.17 (d, 11.2) | |
| 18 | 1.44 (d, 6.8) | 13.1 (q) | 1.59 (d, 6.5) | 13.7 (q) | 1.62 (d, 6.7) | 13.4 (q) |
| 19 | 5.58 (q, 6.8) | 124.8 (d) | 5.43 (q, 6.5) | 122.4 (d) | 5.48 (q, 6.7) | 123.6 (d) |
| 20 | | 140.3 (s) | | 137.7 (s) | | 132.0 (s) |
| 21α | 4.02 (2H, s) | 66.0 (t) | 3.72 (br. d, 17.7) | 46.9 (t) | 3.90 (br. d, 17.5) | 45.9 (t) |
| 21β | | | 3.20 (d, 17.7) | | 3.39 (d, 17.5) | |
| 22 | | | | 173.7 (s) | | 171.9 (s) |
| $NMe_{(1)}$ | 3.59 (s) | 29.3 (q) | | | | |
| $NMe_{(4)}$ | 2.55 (s) | 41.5 (q) | | | | |
| COOMe | | | 3.66 (s) | 52.1 (q) | 3.64 (s) | 51.7 (q) |
| $OMe_{(11)}$ | | | | | 3.45 (s) | 55.0 (q) |
| *CH₃*COO | | | 1.62 (s) | 20.2 (q) | | |
| $CH_3$COO | | | | 171.9 (s) | | |
| $OMe_{(3',5')}$ | | | | | 3.87 (s) | 56.1 (q) |
| $OMe_{(4')}$ | | | | | 3.87 (s) | 60.8 (q) |
| 1′ | | | | | | 124.3 (s) |
| 2′, 6′ | | | | | 6.94 (s) | 106.7 (d) |
| 3′, 5′ | | | | | | 152.5 (s) |
| 4′ | | | | | | 142.0 (s) |
| 7′ | | | | | | 164.6 (s) |

[a]Recorded in $CD_3OD$. [b]Recorded in $CDCl_3$.

reported previously,[15] however, all tested compounds were inactive, and they showed $IC_{50}$ values > 40 $\mu$M.

## Experimental Section

**General Experimental Procedures**. Optical rotations were measured on a Jasco P-1020 automatic digital polarimeter. IR spectra were obtained using a Bruker Tensor 27 FT-IR spectrometer with KBr pellets. NMR spectra were acquired with a Bruker DRX-500 instrument at room temperature. ESIMS (including HRESIMS) were measured on API QSTAR Pulsar i mass spectrometers. Silica gel (200−300 mesh, Qingdao Marine Chemical Inc., China) and Sephadex LH-20 (Amersham Biosciences, Sweden) were used for column chromatography. Fractions were monitored by TLC (Qingdao Marine Chemical Inc., China) in combination with reversed-phase HPLC (Agilent 1200, Extend-C18 column, 5 $\mu$m, 4.6 × 150 mm).

**Plant Material.** The aerial parts of *R. yunnanensis* were collected in Xishuangbanna of Yunnan Province, China, in May 2008 and were identified by Prof. Yu Chen of Kunming Institute of Botany, Chinese Academy of Sciences. The voucher specimen was deposited at BioBioPha Co., Ltd.

**Extraction and Isolation.** The air-dried and powdered aerial parts of *R. yunnanensis* (6.5 kg) were extracted three times with methanol (3 × 50 L, each 3 days) at room temperature and filtered. The filtrate was evaporated under reduced pressure to get a residue (~480 g), which was fractionized by silica gel (200–300 mesh) CC, eluted with a gradient solvent system of petroleum ether-acetone and then MeOH to yield five fractions A–E. Fraction D, eluted by 100% acetone, was separated on silica gel ($CHCl_3$-MeOH, 100:1→30:1) to give two subfractions D1 and D2, Fr. D2 was further isolated and purified by Sephadex LH-20, silica gel ($CHCl_3$-MeOH-ammonia, 100:1:0.1) to afford **3** (28 mg). Fraction E, eluted by 100% methanol, was separated on silica gel ($CHCl_3$-MeOH, 30:1→10:1) to give four subfractions E1–E4, Fr. E4 was further isolated and purified by Sephadex LH-20, and then silica gel ($CHCl_3$-MeOH-ammonia, 30:1:0.1) to afford **1** (40 mg) and **2** (11 mg). The retention times ($t_R$) of **1**–**3** from analysis-type HPLC (50%→100% MeOH in $H_2O$ over 6.0 min followed by 100% MeOH to 10 min, 1.0 ml/min, 20°C) were 5.8, 4.7, and 6.6 min, respectively.

**Rauvoyunine A (1):** yellowish, amorphous powder; $[\alpha]_D^{25}$ + 5.5 (*c* 0.10, MeOH); UV (MeOH) $\lambda_{max}$: 216 (sh), 228, 282, 303, 316 (sh) nm; IR (KBr) $\nu_{max}$ 3432, 2923, 1628, 1467, 1380, 1158, 1134 $cm^{-1}$; $^1H$ and $^{13}C$ NMR data see Table 1; ESIMS (pos.): *m/z* 357 $[M + H]^+$; HRESIMS (pos.): *m/z* 357.2185 (calc. for $C_{21}H_{29}N_2O_3$, 357.2178).

**Rauvoyunine B (2):** yellowish, amorphous powder; $[\alpha]_D^{25}$ –92.0 (*c* 0.19, MeOH); UV (MeOH) $\lambda_{max}$: 231 (sh), 292 nm; IR (KBr) $\nu_{max}$ 3440, 2951, 1738, 1622, 1454, 1385, 1236, 1146, 1114, 1048 $cm^{-1}$; $^1H$ and $^{13}C$ NMR data see Table 1; ESIMS (pos.): *m/z* 427 $[M + H]^+$; HRESIMS (pos.): *m/z* 427.1868 (calc. for $C_{23}H_{27}N_2O_6$, 427.1869).

**Rauvoyunine C (3):** yellowish, amorphous powder; $[\alpha]_D^{25}$ –116.8 (*c* 0.18, MeOH); UV (MeOH) $\lambda_{max}$: 269, 293 (sh) nm; IR (KBr) $\nu_{max}$ 3433, 2945, 1722, 1625, 1590, 1503, 1459, 1384, 1416, 1335, 1226, 1127 $cm^{-1}$; $^1H$ and $^{13}C$ NMR data see Table 1; ESIMS (pos.): *m/z* 593 $[M + H]^+$; HRESIMS (pos.): *m/z* 593.2496 (calc. for $C_{32}H_{37}N_2O_9$, 593.2499).

**Cytotoxicity Assay.** The cytotoxicity assay was performed according to the MTT [3-(4,5-dimethylthiazol-2-yl)-2,5-diphenyltetrazolium bromide] method,[16] by use of the following five human cancer cell lines: human myeloid leukemia HL-60, hepatocellular carcinoma SMMC-7721, lung cancer A-549, breast cancer MCF-7, and colon cancer SW-480. The IC50 values were calculated by Reed and Muench's method.[17]

## Electronic Supplementary Material

Supplementary material is available in the online version of this article at http://dx.doi.org/10.1007/s13659-011-0023-7 and is accessible for authorized users.

## Acknowledgments

This work was financially supported by National Basic Research Program of China (973 Program) 2009CB522300, the "West Light" program of Chinese Academy of Sciences, and Natural Product Library Program of BioBioPha.

**Open Access** This article is distributed under the terms of the Creative Commons Attribution License which permits any use, distribution, and reproduction in any medium, provided the original author(s) and source are credited.

## Electronic supplementary material

Supplementary material, approximately 2.49 MB.
