# Supplementary material for: Three new indole alkaloids from Rauvolfia yunnanensis
Source: Nat Prod Bioprospect. 2011 Dec 1;1(3):104–7. doi: 10.1007/s13659-011-0023-7 (PMC4131643; doi:10.1007/s13659-011-0023-7)

Electronic Supplementary Material

Three new indole alkaloids from *Rauvolfia yunnanensis*

Yuan GAO,[a,b,c] Fei WANG,[a,b] Dong-Sheng ZHOU,[b] Yan LI,[a] and Ji-Kai LIU[a,]*

[a]State Key Laboratory of Phytochemistry and Plant Resources in West China, Kunming Institute of Botany, Chinese Academy of Sciences, Kunming 650201, China

[b]BioBioPha Co., Ltd., Kunming 650201, China

[c]Graduate University of Chinese Academy of Sciences, Beijing 100049, China

Received 11 October 2011; Accepted 23 November 2011

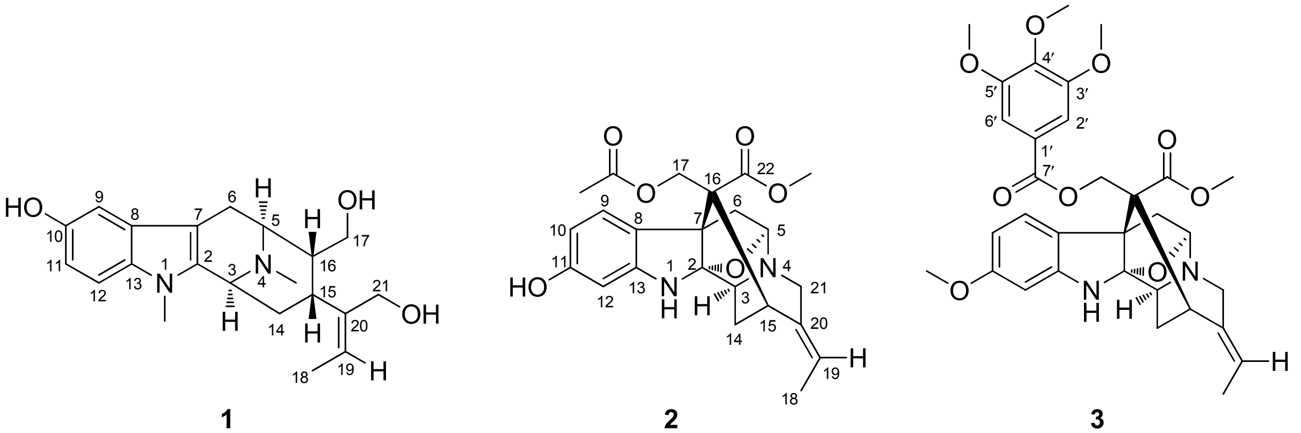

1 **2** **3**

Structures of compounds **1**–**3**.

*To whom correspondence should be addressed. E-mail: jkliu@mail.kib.ac.cn.

Content list:

S1. ^{1}H NMR spectrum (500 MHz, CD_3OD) of rauvoyunine A (**1**).

S2. ^{13}C NMR spectrum (100 MHz, CD_3OD) of rauvoyunine A (**1**).

S3. ^{13}C NMR (DEPT) spectrum (100 MHz, CD_3OD) of rauvoyunine A (**1**).

S4. HMBC spectrum (500 MHz, CD_3OD) of rauvoyunine A (**1**).

S5. ROESY spectrum (400 MHz, CD_3OD) of rauvoyunine A (**1**).

S6. ^{1}H NMR spectrum (500 MHz, CD_3OD) of rauvoyunine B (**2**).

S7. ^{13}C NMR spectrum (100 MHz, CD_3OD) of rauvoyunine B (**2**).

S8. ^{13}C NMR (DEPT) spectrum (100 MHz, CD_3OD) of rauvoyunine B (**2**).

S9. HMBC spectrum (500 MHz, CD_3OD) of rauvoyunine B (**2**).

S10. ROESY spectrum (600 MHz, CD_3OD) of rauvoyunine B (**2**).

S11. ^{1}H NMR spectrum (400 MHz, $CDCl_3$) of rauvoyunine C (**3**).

S12. ^{13}C NMR spectrum (125 MHz, $CDCl_3$) of rauvoyunine C (**3**).

S13. ^{13}C NMR (DEPT) spectrum (125 MHz, $CDCl_3$) of rauvoyunine C (**3**).

S14. HMBC spectrum (500 MHz, $CDCl_3$) of rauvoyunine C (**3**).

S15. ROESY spectrum (600 MHz, $CDCl_3$) of rauvoyunine C (**3**).

S1. ^{1}H NMR spectrum (500 MHz, CD_3OD) of rauvoyunine A (**1**).

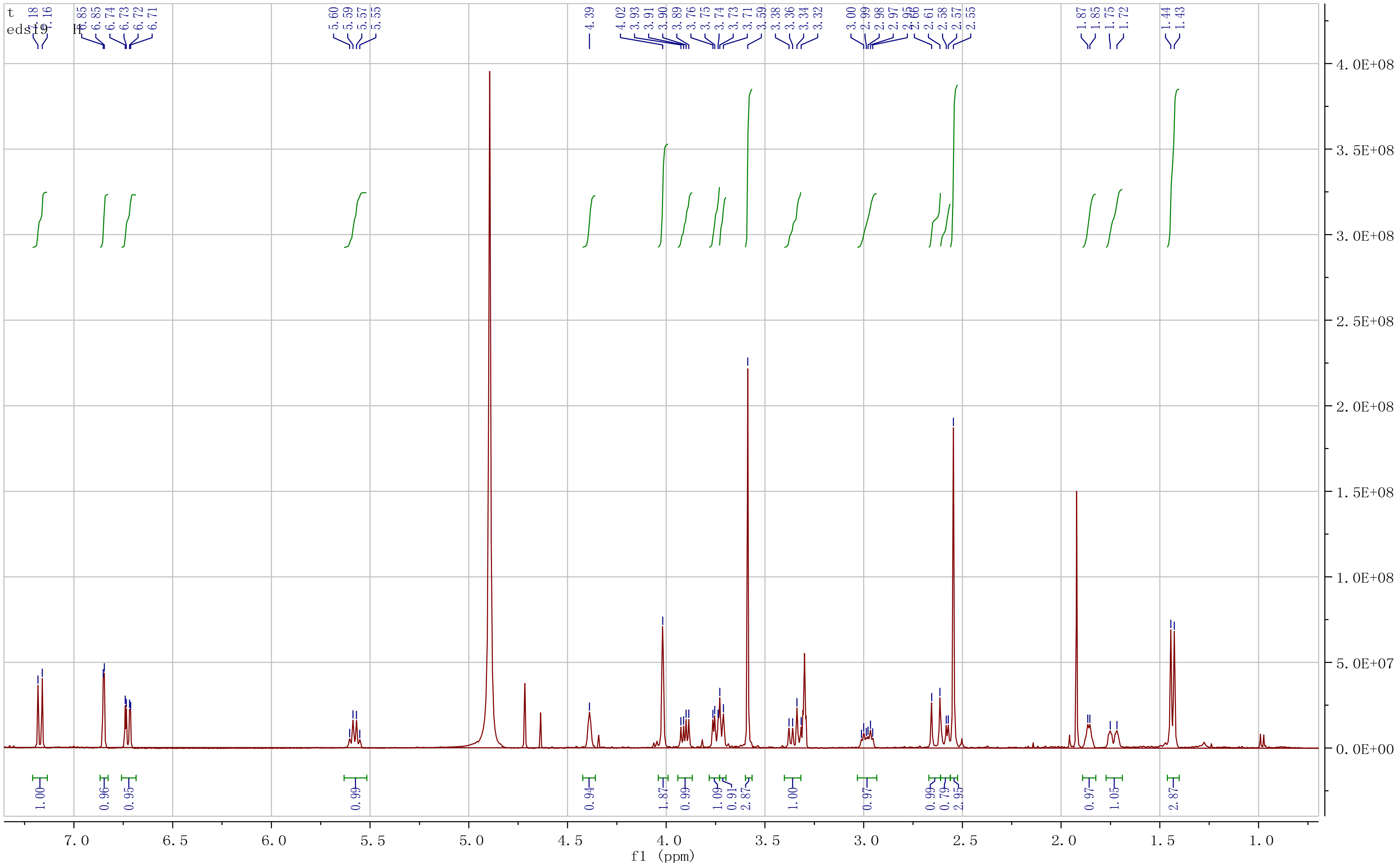

S2. ^{13}C NMR spectrum (100 MHz, CD_3OD) of rauvoyunine A (**1**).

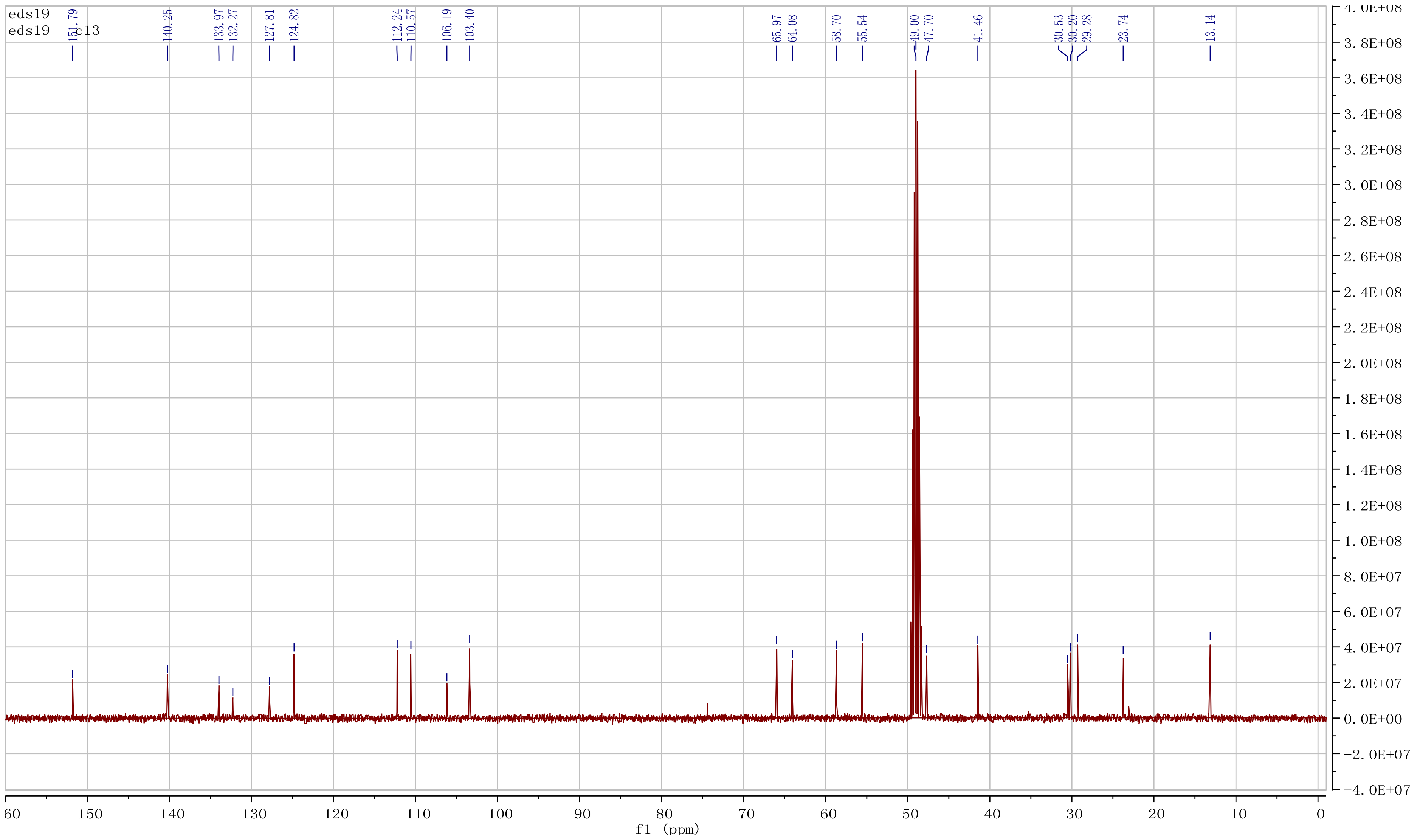

S3. ^{13}C NMR (DEPT) spectrum (100 MHz, CD_3OD) of rauvoyunine A (**1**).

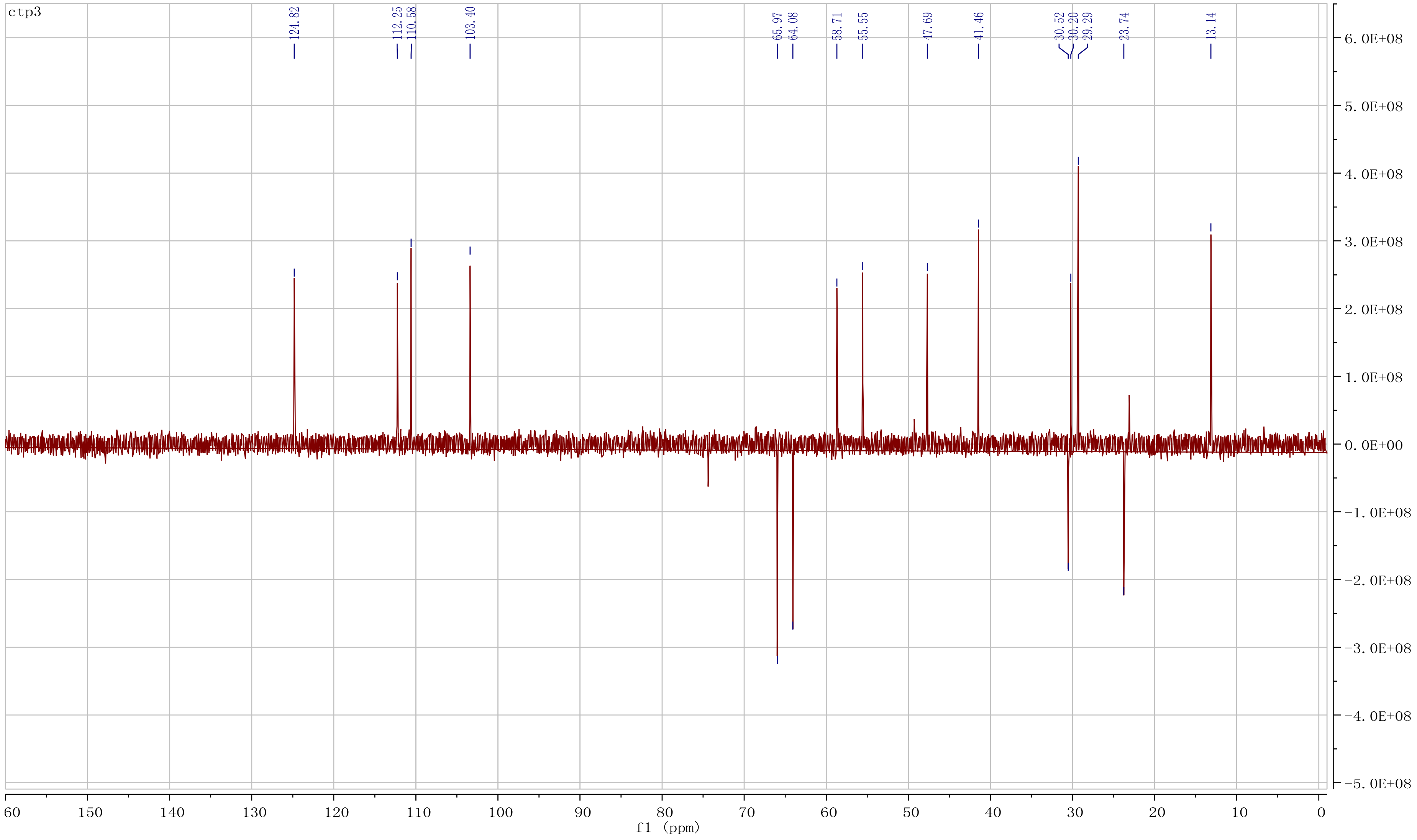

S4. HMBC spectrum (500 MHz, CD_3OD) of chloranthiolide rauvoyunine A (**1**).

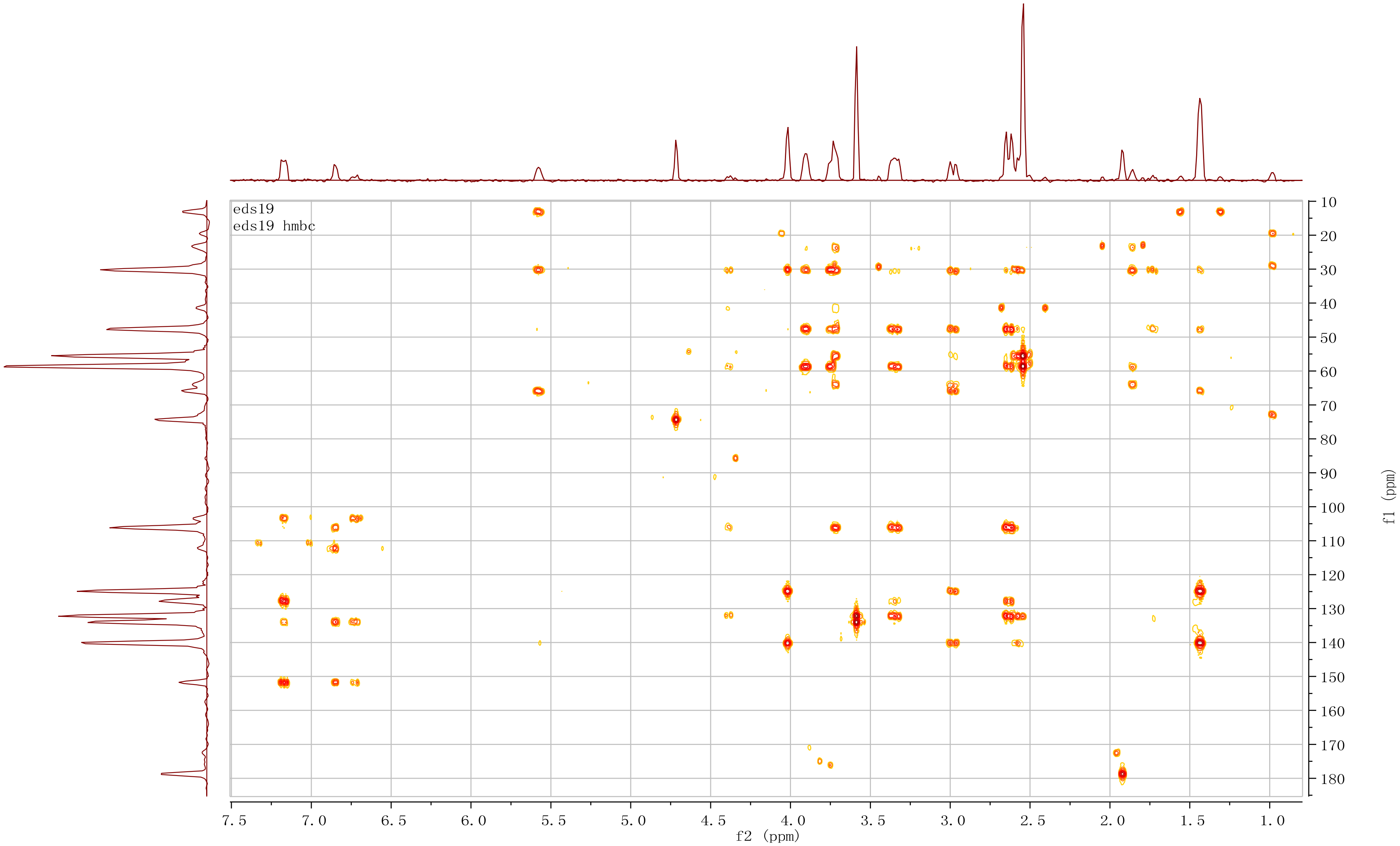

S5. ROESY spectrum (400 MHz, CD_3OD) of chloranthoLide rauvoyunine A (**1**).

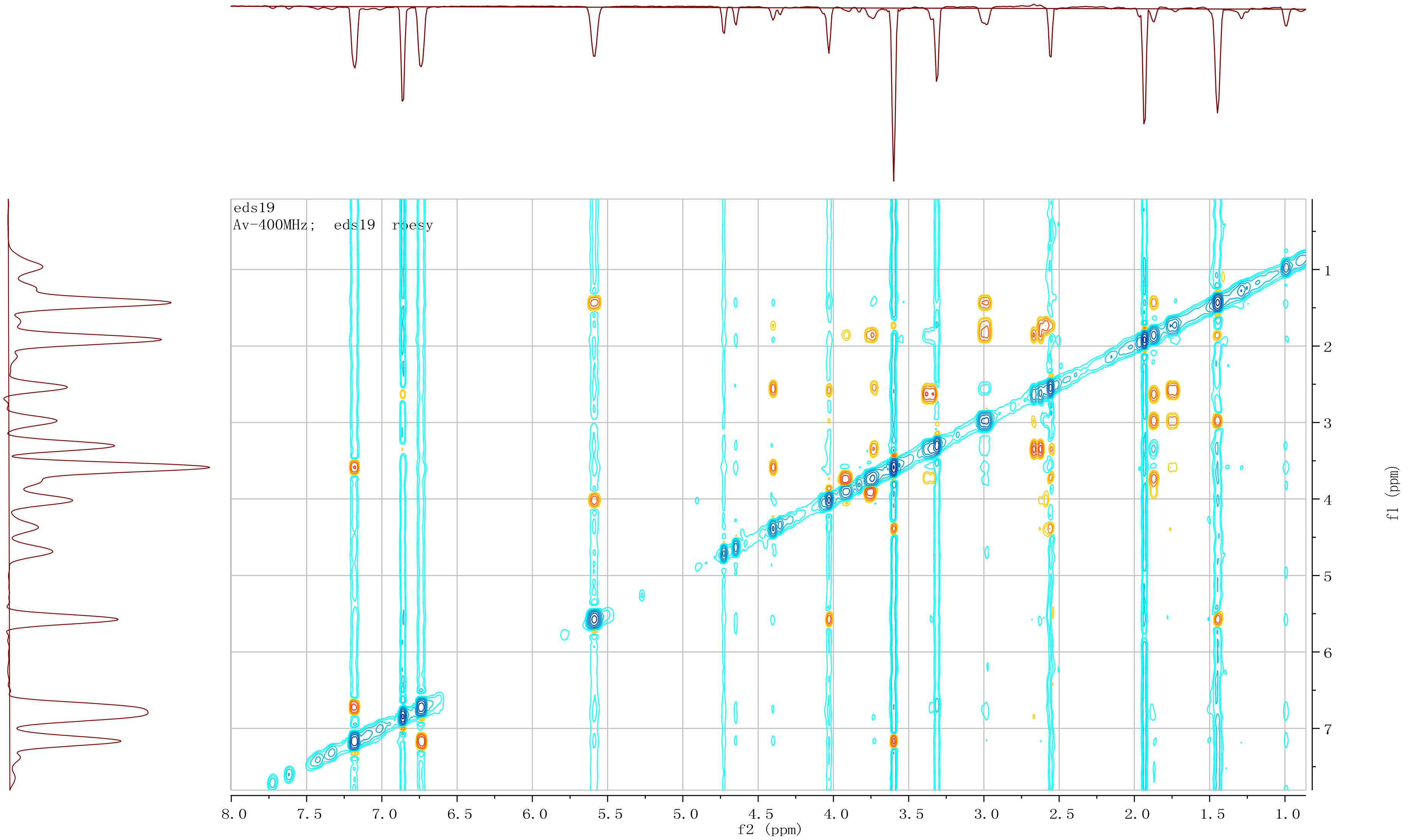

S6. ^{1}H NMR spectrum (500 MHz, CD_3OD) of rauvoyunine B (**2**).

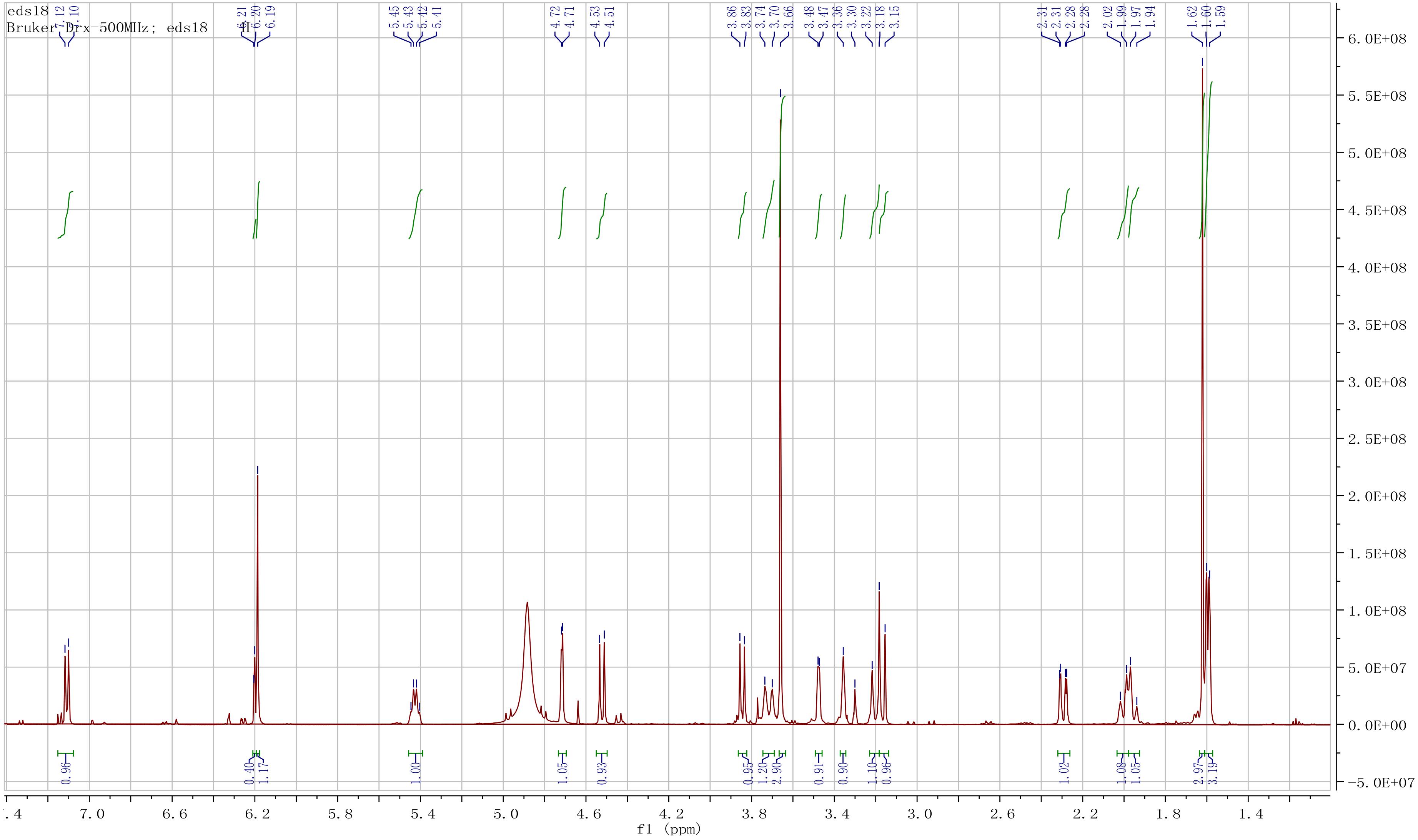

S7. ^{13}C NMR spectrum (100 MHz, CD_3OD) of rauvoyunine B (**2**).

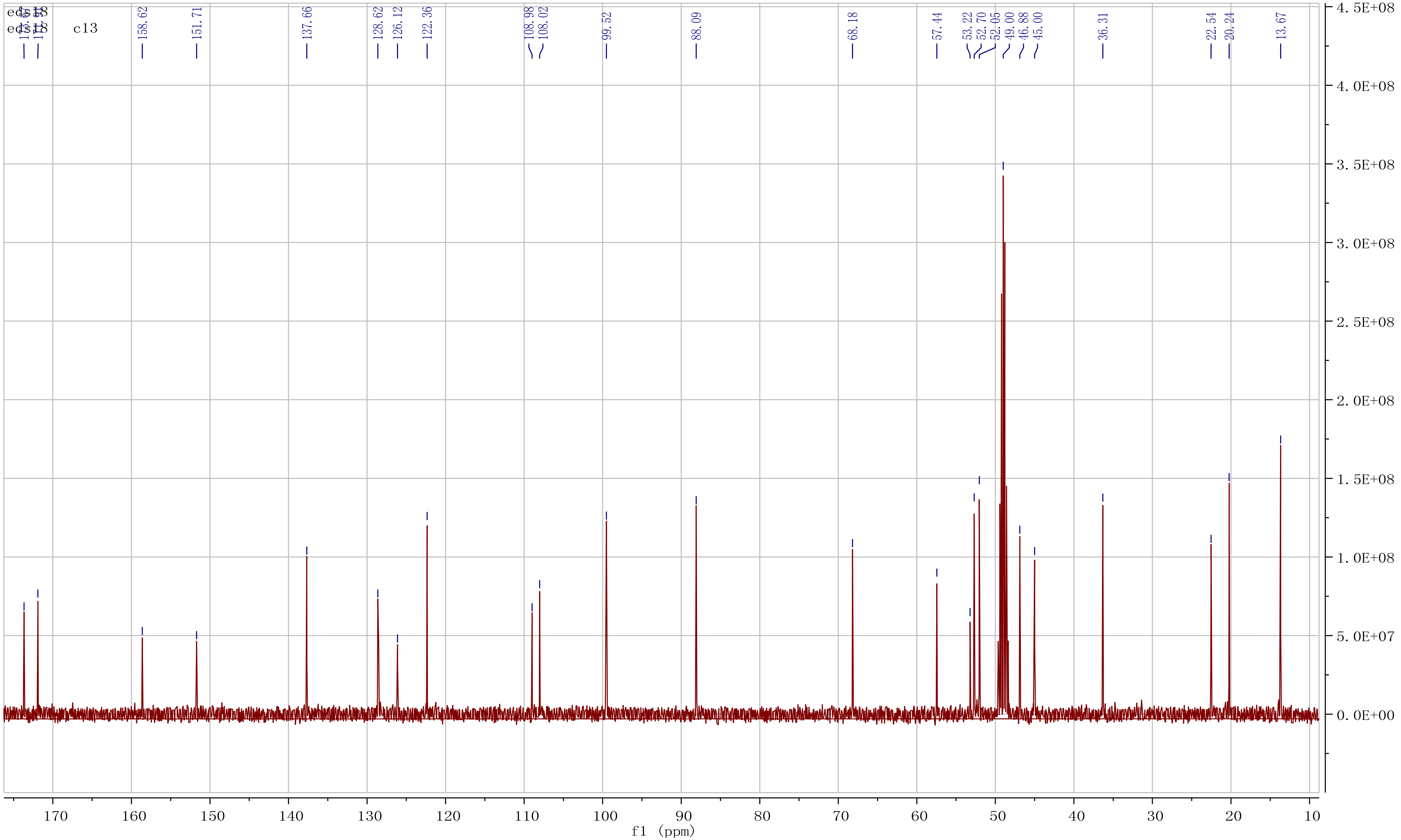

S8. ^{13}C NMR (DEPT) spectrum (100 MHz, CD_3OD) of rauvoyunine B (**2**).

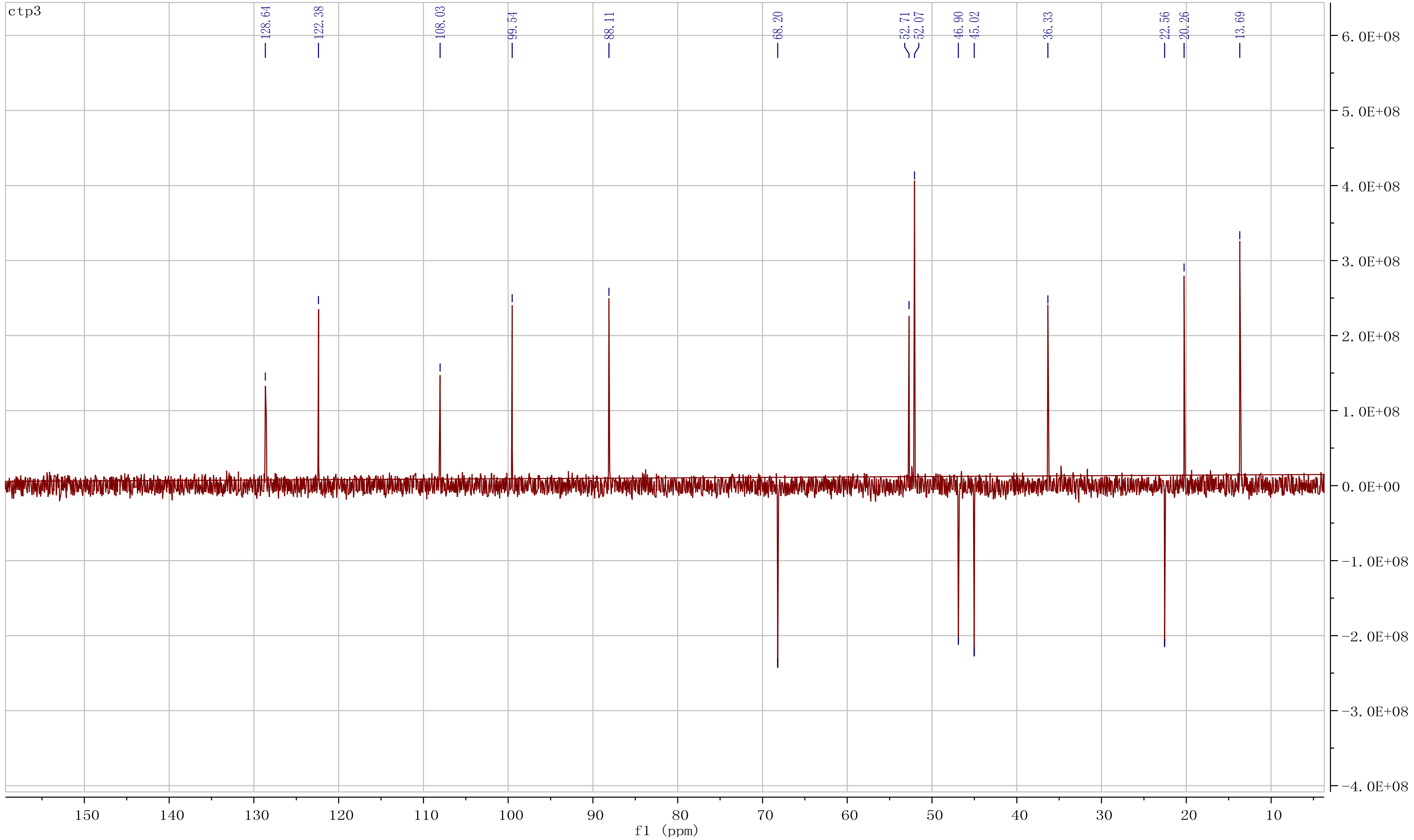

S9. HMBC spectrum (500 MHz, CD_3OD) of rauvoyunine B (**2**).

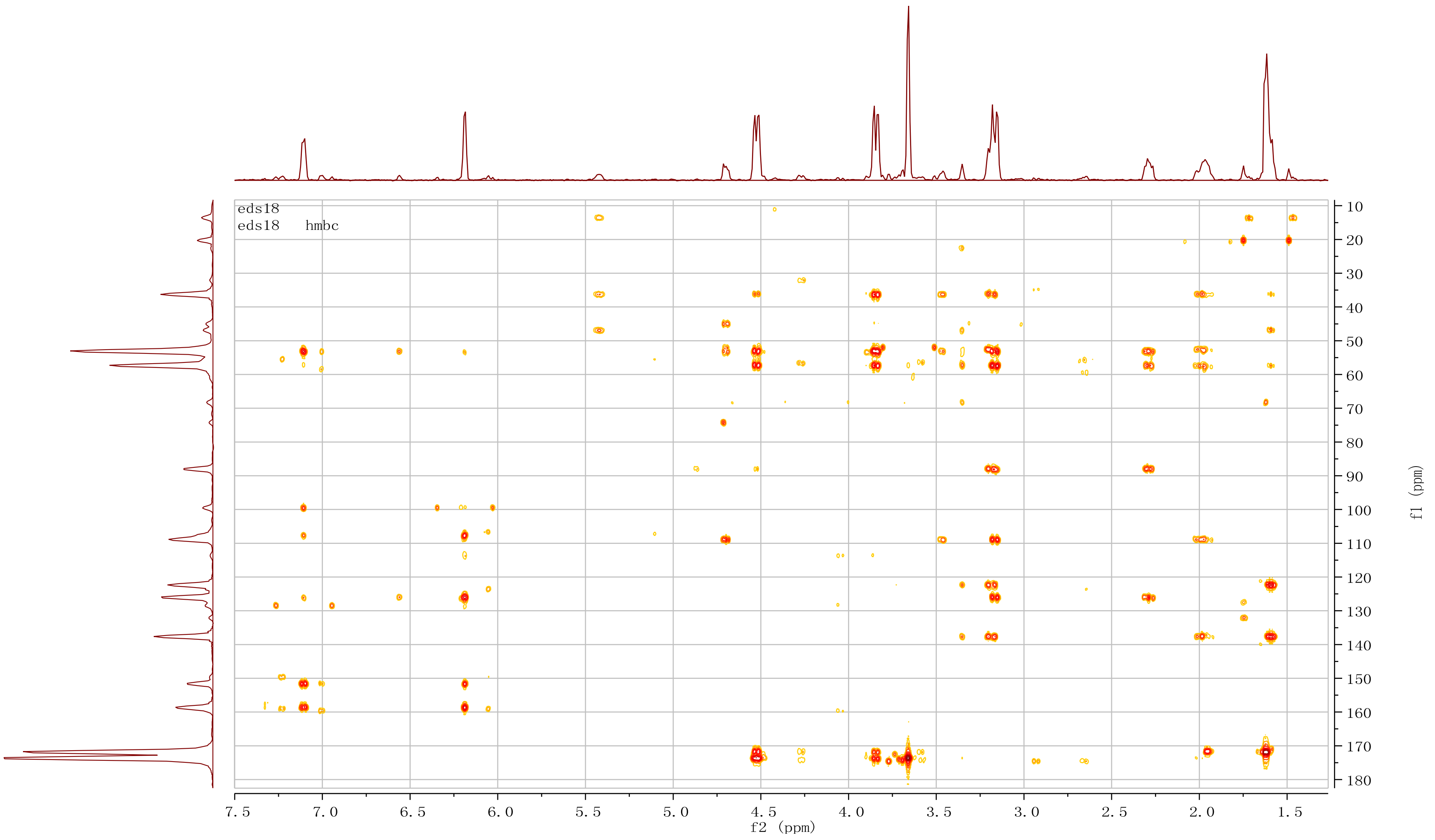

S10. ROESY spectrum (600 MHz, CD_3OD) of rauvoyunine B (**2**).

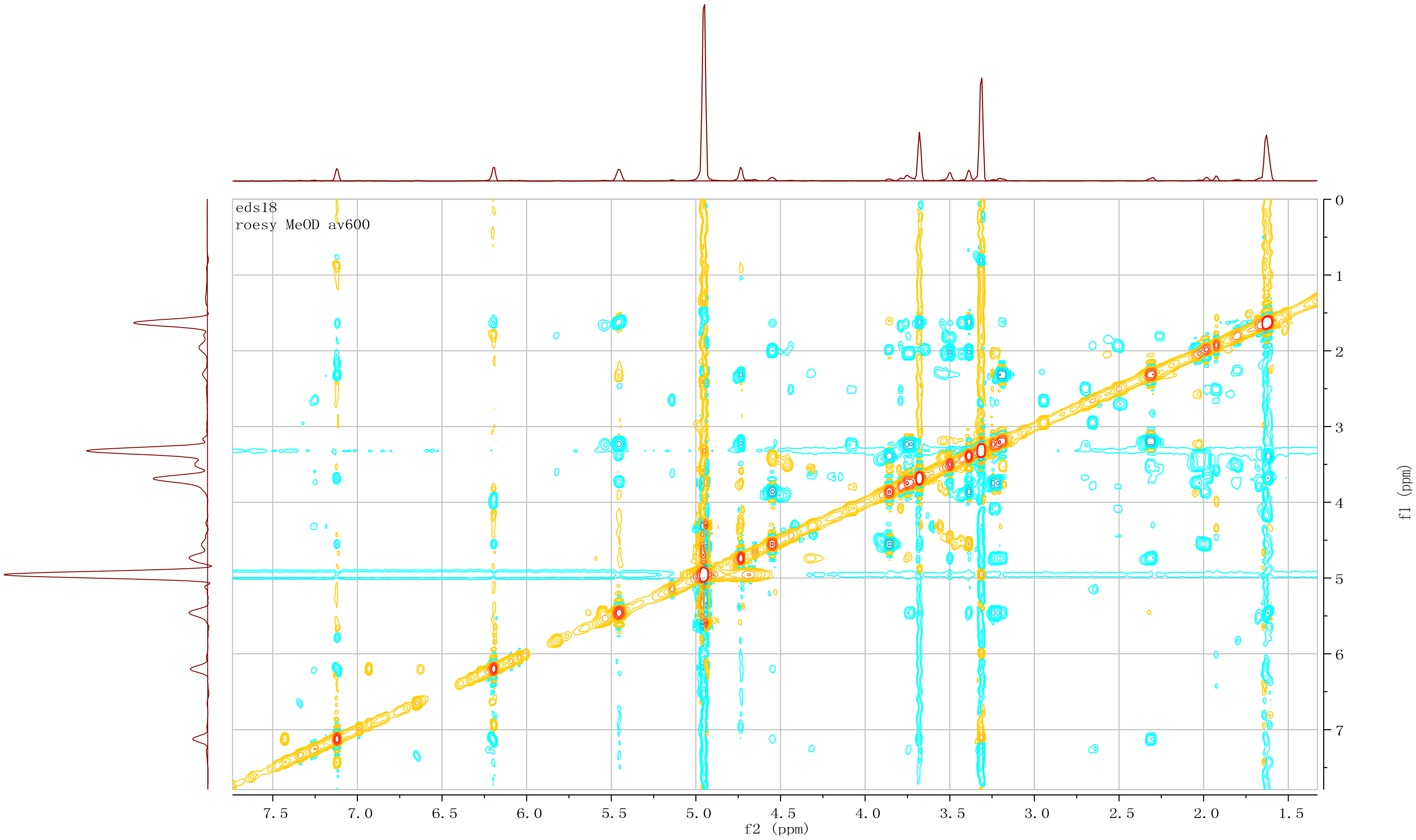

S11. ^{1}H NMR spectrum (400 MHz, $CDCl_3$) of rauvoyunine C (**3**).

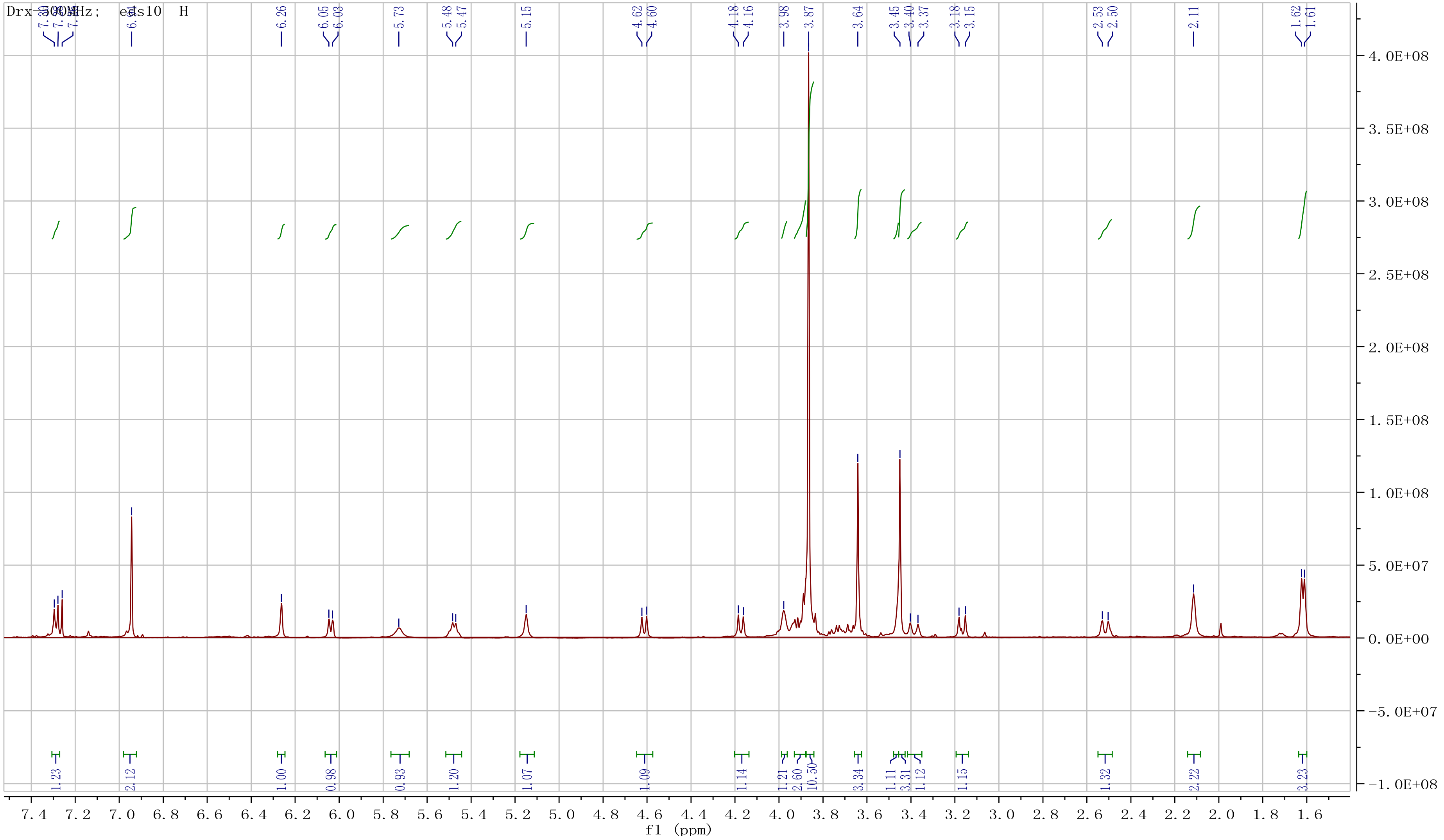

S12. ^{13}C NMR spectrum (125 MHz, $CDCl_3$) of rauvoyunine C (**3**).

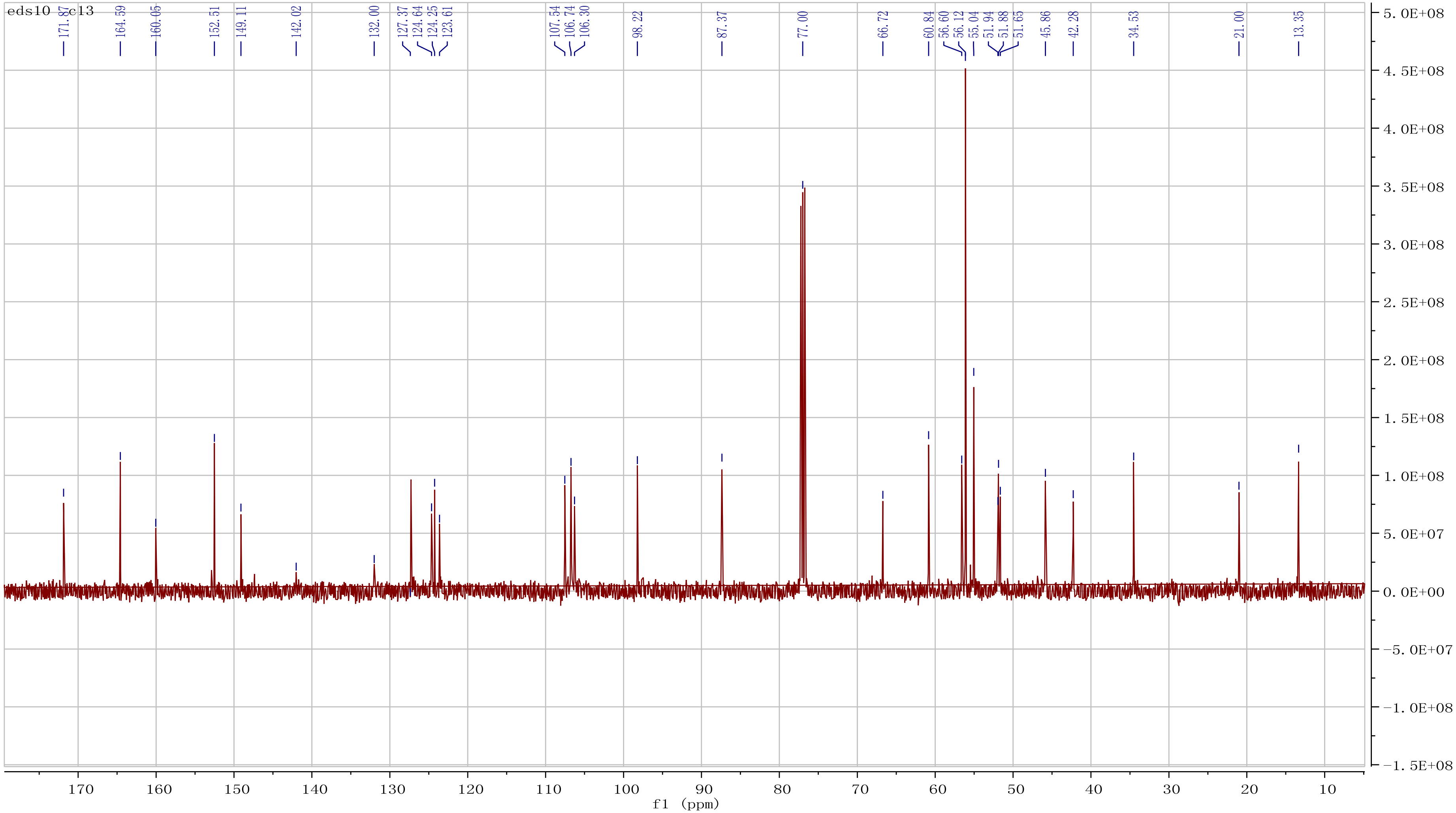

S13. ^{13}C NMR (DEPT) spectrum (125 MHz, $CDCl_3$) of rauvoyunine C (**3**).

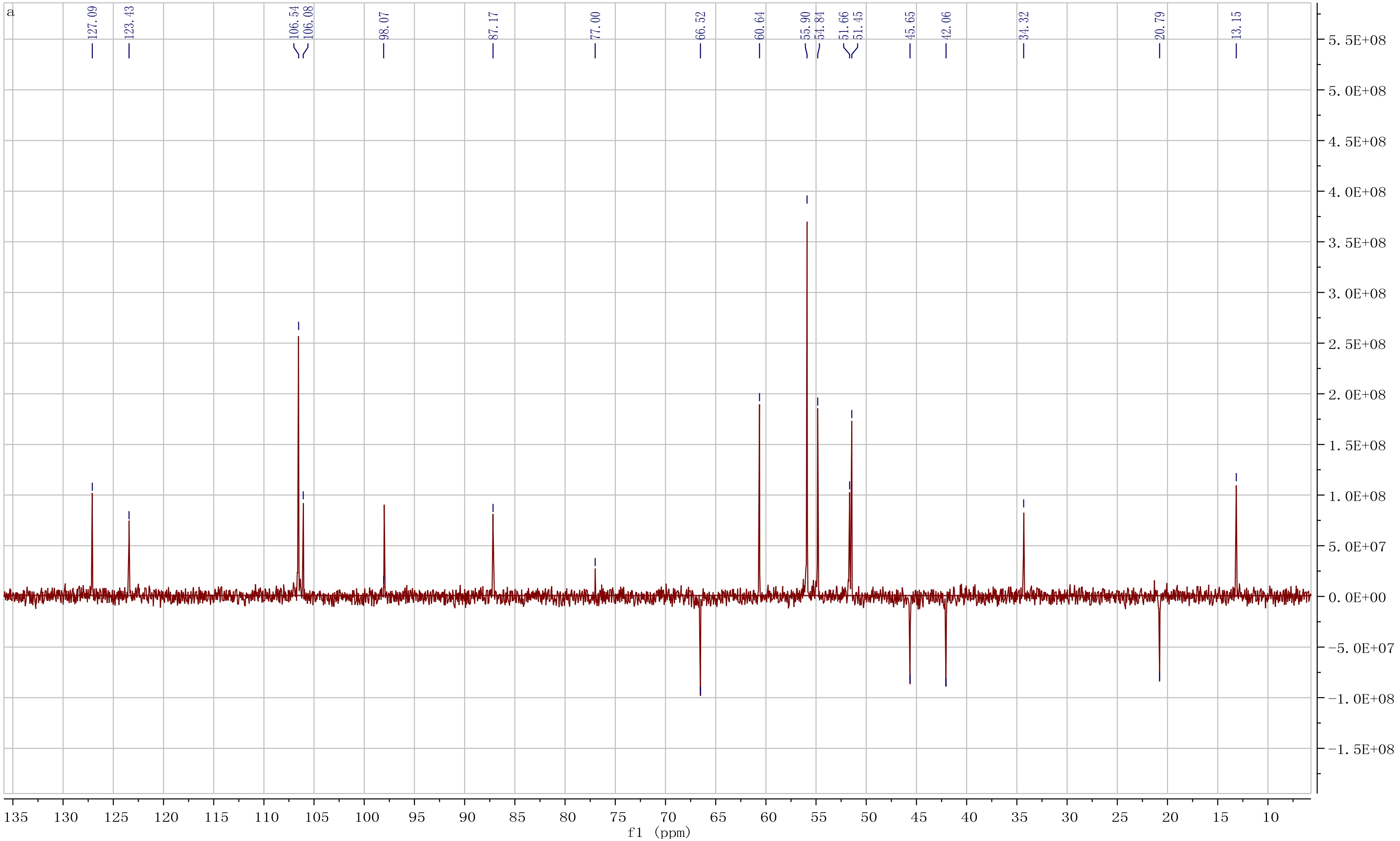

S14. HMBC spectrum (500 MHz, $CDCl_3$) of rauvoyunine C (**3**).

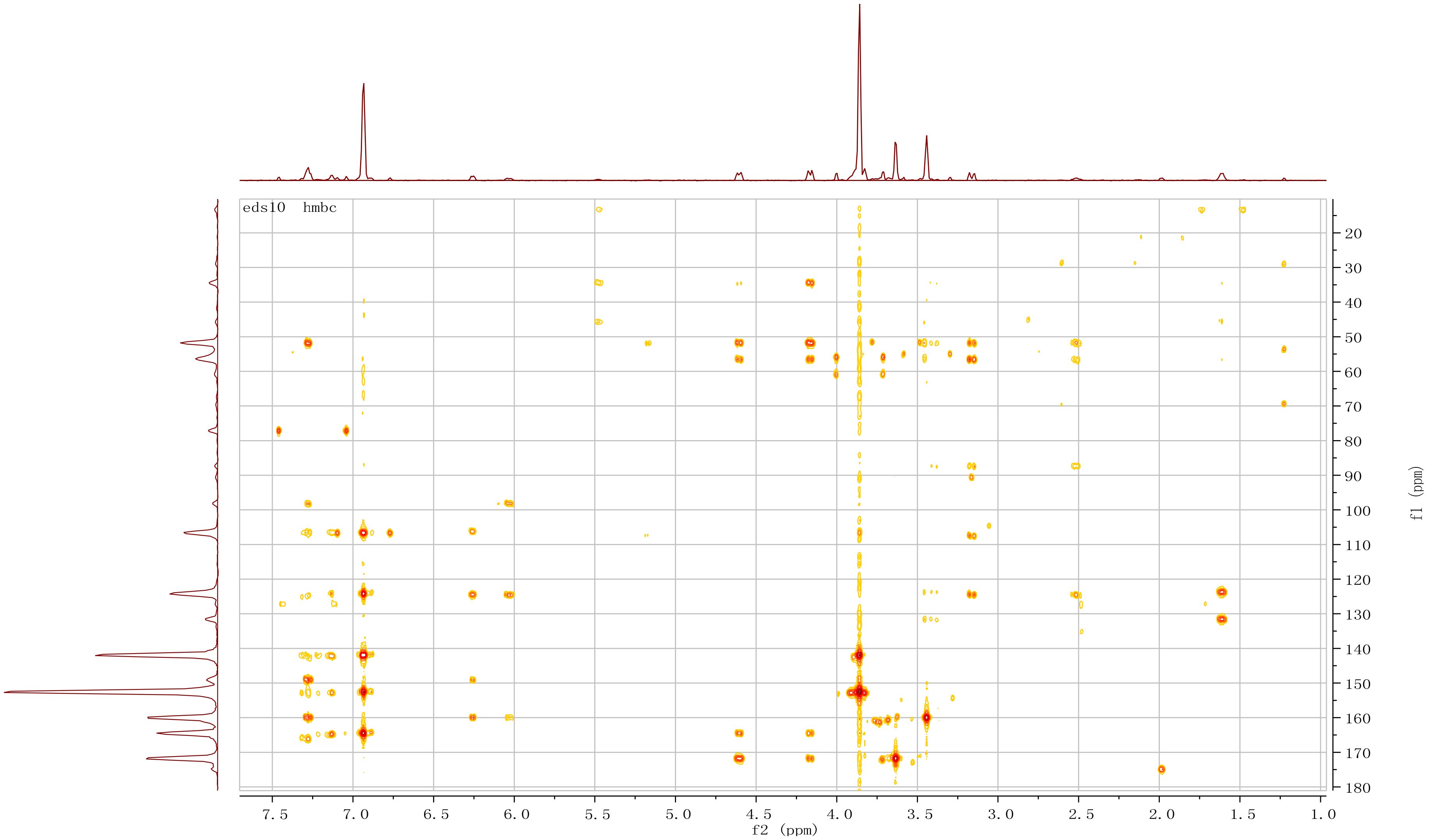

S15. ROESY spectrum (600 MHz, $CDCl_3$) of rauvoyunine C (**3**).

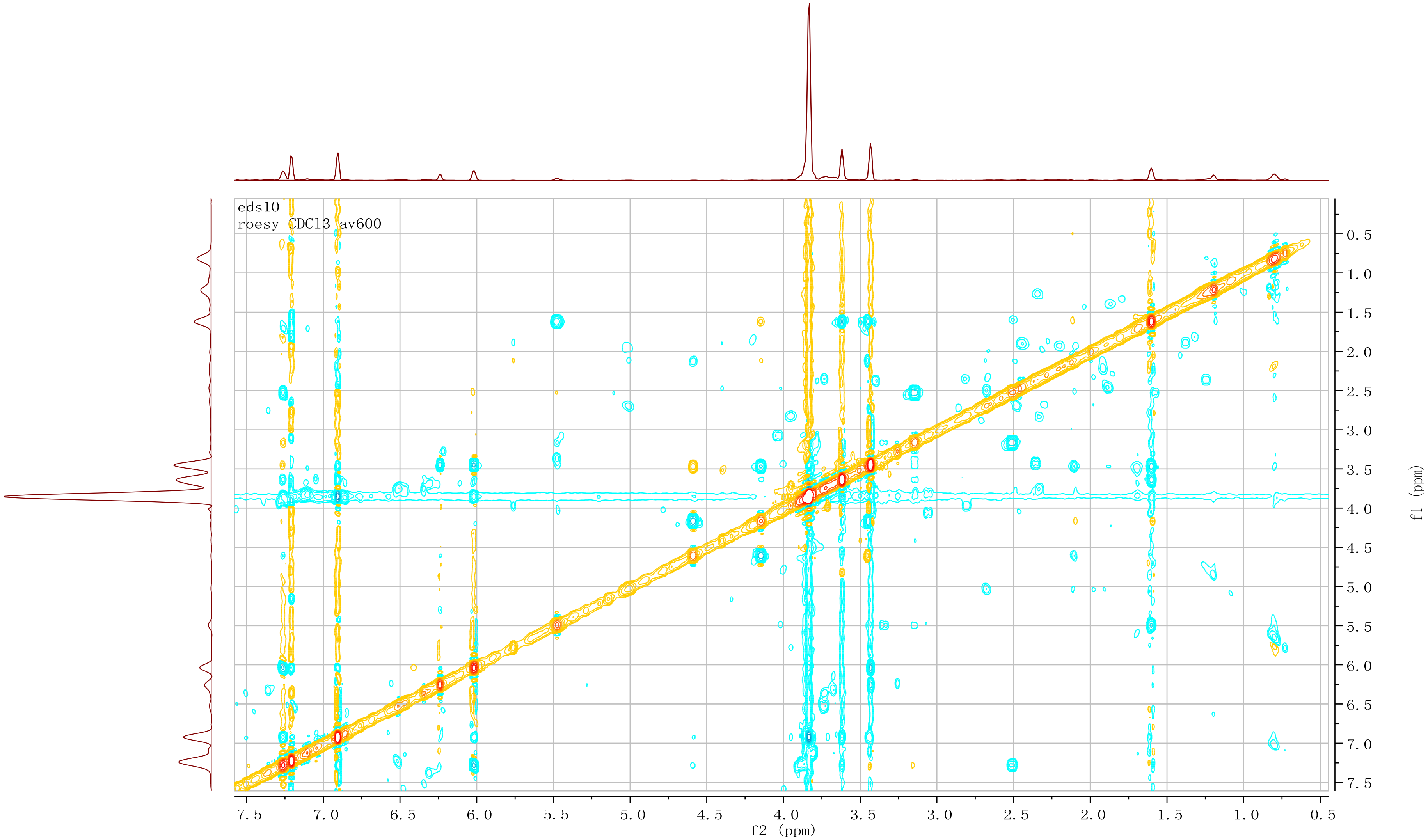

Supplement: Supplementary file 1 — Supplementary material, approximately 2.49 MB. [file 13659_2011_23_MOESM1_ESM.pdf]
